# Yiqihuoxue decoction protects against post-myocardial infarction injury via activation of cardiomyocytes PGC-1α expression

**DOI:** 10.1186/s12906-018-2319-1

**Published:** 2018-09-17

**Authors:** Fanghe Li, Shuwen Guo, Chunguo Wang, Xiaolou Huang, Hui Wang, Xiaobo Tan, Qian Cai, Jiani Wu, Yuqin Zhang, Xi Chen, Wangou Lin, Binyue Zhang

**Affiliations:** Beijing University of Chinese Medicine, Beijing, 100029 China

**Keywords:** Yiqihuoxue decoction (YQHX), Myocardial ischemia, Cardiomyocytes, PGC-1α

## Abstract

**Background:**

Mitochondrial dysfunction has been implicated in the pathogenesis of ischemic heart disease, exacerbating cardiomyocytes injury in myocardial infarction (MI). Peroxisome proliferator-activated receptor gamma co-activator (PGC-1α) has been recognized as the key regulator of mitochondrial biogenesis and energy metabolism. Yiqihuoxue decoction (YQHX), a Traditional Chinese Medicine (TCM) prescription, can prevent and treat ischemic heart disease. However, the mechanisms of YQHX on PGC-1α expression in the ischemic heart have remained unclear.

**Methods:**

Myocardial ischemia rat model and ischemia/hypoxia injury model in the cardiomyocytes were used to minic human cardiovascular disease. Rats were randomly assigned into 4 groups: Sham, Model, YQHX (8.2 g/kg) and Trimetazidine (10 mg/kg) group. 28 days after MI, cardiac functions and morphology were detected by echocardiography and HE staining, respectively. In vitro, the effects of YQHX on H9c2 cell viability, LDH and ROS were detected, respectively. PGC-1α relevant proteins were evaluated by Western blotting.

**Results:**

In vivo, echocardiography and HE staining results showed that YQHX improved cardiac functions and modified pathological changes. YQHX enhanced PGC-1α expression and improved the mitochondrial ultrastructure and functions in rats MI model for 4 weeks. Further, we explored its potential mechanisms in cardiomyocytes. In vitro, YQHX significantly enhanced cell viability and reduced LDH release and ROS production induced by hypoxia in cardiomyocytes. Interestingly, exposure of cardiomyocytes to hypoxic conditions for 12 h induced the downregulation of PGC-1α expression, but the expression levels nearly returned to the normal state after hypoxia for 24 h. YQHX significantly enhanced PGC-1α expression between 12 h and 24 h induced by hypoxia through a mechanism associated with the activation of AMPK phosphorylation in H9c2 cells. In addition, YQHX upregulated the expression of Tfam and NRF-1, while NRF-1 expression was completely blocked by an AMPK inhibitor. YQHX largely restored the mitochondrial morphology and increased mitochondrial membrane potential in hypoxia-induced injury. Furthermore, the UHPLC-LTQ-Orbitrap-MS^n^ analysis found that there were 87 chemical constituents in YQHX.

**Conclusions:**

These results suggest that the protective effect of YQHX on cardiomyocytes against hypoxia-induced injury may be attributed to activation of PGC-1α and maintenance of mitochondrial functions through a mechanism involving the activation of AMPK phosphorylation.

**Electronic supplementary material:**

The online version of this article (10.1186/s12906-018-2319-1) contains supplementary material, which is available to authorized users.

## Background

Ischemic heart disease (IHD), a crucial common cardiovascular disease, is characterized by reduced blood supply to the heart which leads to cardiomyocytes loss, ventricular remodeling, and even heart failure [1]. IHD is a public health problem with high morbidity and mortality [2]. Indeed, epidemiological studies have reported that IHD ranks as the second leading causes of death in China in 2016 [3]. Myocardial infarction (MI) is the primary pathologic complication in IHD caused by coronary artery obstruction. Although the rapid restoration of coronary artery blood flow can limit the infarct size and eventually improve the survival of MI patients [4, 5], this intervention does not adequately prevent the deterioration of cardiac function as it is associated with reperfusion injury. Therefore, additional research is required to develop efficient therapeutic options for the clinical treatment of IHD.

It is known that the mitochondria are the powerhouses of cardiomyocytes. After myocardial infarction, a series of mitochondrial alterations occur in the heart, including alterations in mitochondrial morphology and biogenesis [6], excessive accumulation of reactive oxygen species (ROS) [7] and the opening of the mitochondrial membrane potential [8]. Therapeutic measures targeting the mitochondria have been recognized to prevent and treat the cardiovascular diseases. Peroxisome proliferator-activated receptor gamma (PPARγ) co-activator (PGC-1α), a member of the PGC-1 family [9], has been recognized as a central regulator of mitochondrial biogenesis and energy metabolism [10]. PGC-1α is a regulator of mitochondrial biogenesis via activating nuclear respiratory factor 1 (NRF-1) and the synthesis of the transcription factor A mitochondrial (Tfam) [11]. The PGC-1α activity can be modulated by AMP-activated protein kinase (AMPK) phosphorylation [12]. It has also been associated with a wide range of biological processes. There is increasing evidence that PGC-1α exerts different effects on cell metabolism depending on the type of organs. For instance, Aatsinki et al. [13] reported that PGC-1α activated gluconeogenesis in hepatocytes, while Ye et al. [14] showed that PGC-1α prevented hypoxia-induced pulmonary artery endothelial dysfunction. Thus, there has been substantial interest to understand how PGC-1α becomes downregulated during myocardial infarction.

Yiqihuoxue Decoction (YQHX) is designed based on the well-known Traditional Chinese Medicines (TCMs) formula Danggui Buxue decoction (DBD). DBD, consisting of *Astragalus membranaceus* and *Angelica sinensis*, has been first described in 1247 A.D. by Dong-Yuan Li in < Neiwaishangbianhuolun > [15]. DBD has been used for nourishing “qi” and enriching “blood” to prevent and treat cardiovascular diseases such as myocardial infarction [16, 17]. Compound *Astragalus* and *Angelica* extract also were found to promote angiogenesis via regulating VEGFR1/2 and SEGFR1/2 expressions in myocardial infarction rat [16]. Based on the theory of tonifying “qi” and activating “blood” using Danggui Buxue decoction, YQHX is widely used in the prevention and treatment of cardiovascular disease by promoting angiogenesis, inhibiting inflammatory response, and regulating left ventricular function and energy metabolism [18–21]. In myocardial infarction rats model, our previous studies showed that YQHX promoted angiogenesis by the up-regulation of vascular endothelial growth factor expression after 28 days [19]. YQHX was shown to regulate the metabolic-related products such as lipids, amino acids and glycolipids using nuclear magnetic resonance metabolomics [20]. YQHX could ameliorate the cardiac energy metabolism via cross-talk between the LKB1-dependent Notch1 and AMPK after myocardial infarction [21]. Also, YQHX could prevent and treat post-MI myocardial remodeling through regulating left ventricular function and promoting the expression of AMPK signal pathway [22]. However, whether YQHX influences the expression of PGC-1α in the ischemic myocardium, especially in H9c2 cardiomyocytes, remains unclear. Therefore, we aimed to investigate the effects and the mechanisms of YQHX on PGC-1α expression and its cardioprotective effects in vitro and in vivo. This study will provide further insight into the mechanisms of TCM in the prevention and treatment of IHD.

## Methods

### YQHX preparation

YQHX is composed of five medicinal herbs: *Astragalus membranaceus*, *Angelica sinensis*, *Panax ginseng*, *Ligusticum wallichii*, and *Panax notoginseng*. The herbs were purchased from the Dongzhimen Hospital in China. The crude components of YQHX were extracted by refluxing with boiling distilled water (1:10, g/mL), each three times. After filtration, the water extracts were concentrated to a constant volume. The concentrated extracts were used for animal experiments and were prepared in the form of powders by freeze-drying in a vacuum to be used for cell experiments. A portion of each filtrate was diluted 4-fold with distilled water and further subjected to UHPLC-LTQ-Orbitrap-MS.

### HPLC-linear ion trap-Orbitrap mass spectrometry

Chromatographic separation was performed on an Agilent Zorbax SB-C_18_ column (4.6 mm × 250 mm, 5 μm), and the temperature was settled at 25 °C. The flow rate was 1.0 mL/min, the UV spectra were 254 nm and the mobile phases were a mixture of ultrapure water (A) and acetonitrile (B). The following gradient program was used: 0–10 min, 2% B; 10–15 min, 2–5% B; 15–52 min, 5–42% B; 52–70 min, 42–90% B; 70–75 min, 90–90% B; 75–80 min, 90–5% B. High-resolution mass spectra were analyzed using an LTQ-Orbitrap mass spectrometer (Thermo Scientific, Bremen, Germany), which was operated in the negative ionization mode. The parameters were as follows: sheath gas flow rate, 30 arb; aux gas flow rate, 10 arb; capillary temperature, 350 °C; ion spray voltage, 4.0 kV; capillary voltage, − 25 V; and tube lens voltage, − 110 V. In the full scan mode, mass spectra were recorded in the mass range of *m/z* 150 to 1200 with a resolution of 30,000 (full width at half-maximum, as defined at *m/z* 400).

### Animal study

In accordance with the Guide for the Animal Care and Use of Laboratory Animals published by the National Institutes of Health (NIH Publications No. 85–23, revised 1996), all experimental protocols and animal handling procedures were approved by the Animal Care and Use Committee of Dongzhimen Hospital Affiliated to Beijing University of Chinese Medicine (2017–11). Adult male Sprague-Dawley (SD) rats (180–200 g) were obtained from Beijing-Vital-River-Laboratory-Animal Technology (license number: SCXK2016–0011) and were subjected to myocardial infarction surgery. Rats were allowed free access to regular diet and distilled water, and were housed in an air-conditioned animal room at the Key Laboratory of Dongzhimen Hospital before surgery (temperature: 21 ± 2 °C, humidity: 50 ± 5%). The myocardial infarction rat model was induced as described previously [20, 23, 24]. Firstly, the rats were anesthetized with 1% pentobarbital sodium 40 mg/kg by intraperitoneal injection. The chest of the rat was opened by left thoracotomy to expose the heart, and the left anterior descending (LAD) coronary artery was ligated using a 5–0 nylon suture in the MI group and then the chest was closed. The procedure was the same in the Sham group except the LAD ligation. The success of MI model was confirmed by significant elevation of the ST segment arch obtained by monitoring the electrocardiogram (ECG) limb lead (Medical electronic instrument factory, Jiangsu, China). After four weeks, the rats that survived were subjected to echocardiographic examination and those with left ventricular ejection fraction (LVEF) of less than 50% were chosen for subsequent studies. All animals were anaesthetized by pentobarbital sodium, and then cardiac samples were collected to analyze protein expression levels. After that, the rats were euthanized by cervical dislocation. The thumb and index finger of researcher was pressed at the base of the rat’s skull. On the other hand, the base of the tail was rapidly pulled, causing a separation of the cervical vertebrae from the skull.

The experimental animals were randomly divided into four groups (*n* = 12, 8-week-old): Sham operation + vehicle (Sham), Myocardial infarction model + vehicle (MI), Myocardial infarction model + YQHX (YQHX), Myocardial infarction model + Trimetazidine (TMZ). The YQHX was administered at the dosage of 8.2 g/kg/d and the dosage of TMZ (Servier pharmaceutical Co., Ltd., Tianjin, China) was 10 mg/kg/d by irrigation on the second day after MI surgery, as described before [25].

### Cell culture and treatment

H9c2 cells were seeded and cultured in high-glucose DMEM (Invitrogen, California, USA) with 10% fetal bovine serum FBS, 1% penicillin and streptomycin in a cell incubator with 5% CO_2_ and 95% O_2_ at 37 °C. The medium was completely replaced with serum-free DMEM. After synchronization of the culture for 6 h, the control group (C) was cultured in a normoxic atmosphere and the ischemia/hypoxia injury model (I/H) in H9c2 cells was induced by exposure of the cells to a hypoxic atmosphere with 1% O_2_, 5% CO_2_ and 94% N_2_ for 12 h or 24 h. 25 mg of YQHX were dissolved in 50 mL of DMEM and used for the treatment of hypoxia-induced H9c2 cells [26]. Thereafter, the YQHX extracts were filtered through a 0.45 μm millipore filter prior to use. In the rescue groups, the H9c2 cells were treated with YQHX at the concentrations of 100 μg/mL (Y1), 200 μg/mL (Y2) and 400 μg/mL (Y4), and Compound C (5 μM) (Selleck, Shanghai, China) with YQHX (Y2cc) before the initiation of hypoxia.

### Histologic examination and echocardiography

Cardiomyocytes architectures were visualized using hematoxylin and eosin (HE) staining. Rat heart tissues were fixed in 4% paraformaldehyde for 24 h and then were embedded in paraffin. The tissue blocks were cut into 4 μm sections, which were stained with HE to observe the morphology.

Cardiac functions such as the left ventricular internal diastolic diameter (LVIDd), and internal systolic diameter (LVIDs), ejection fraction (EF) and fractional shortening (FS) were measured by echocardiography at 4 weeks after surgery using a 30-MHz high-resolution Vevo 770 small-animal ultrasonic instrument probe (Visual Sonics Inc., Toronto, Canada).

### Measurement of ATP levels

The fresh samples were homogenized in cold lysis solution for 30 min. The lysate was centrifuged at 12,000 g and 4 °C for 5 min to obtain the supernatant. The ATP levels were detected by ATP Assay Kits (Beyotime, Shanghai, China) according to the manufacturer’s protocol.

### Cell viability assay

H9c2 cells were seeded and cultured in 96-well plates. Cell viability was examined using CCK8 assay kit. H9c2 cells were incubated with the cell counting kit-8 (CCK8, Dojindo Laboratories, Kyushu, Japan) for 2 h at 37 °C and then cell viability was measured at 450 nm by a microplate reader. The cell viability was calculated as the percentage of absorbance to control values.

### LDH assay

To further assess the cell injury, the release of lactate dehydrogenase (LDH) was tested by LDH assay. The level of LDH in H9c2 cell supernatants was measured using LDH commercial kit, according to the manufacturer’s protocol (Jian Cheng Bioengineering Institute, Nanjing, China). LDH value was examined using a microplate reader at 440 nm and was expressed as U/L.

### ROS measurement

H9c2 cells were washed three times with phosphate buffered saline (PBS), and then incubated with PBS and 10 μM fluorescent probe 2′, 7′-dichlorofluorescein diacetate (DCFH-DA) in the dark for 30 min at 37 °C. Then the level of ROS was estimated by fluorescence microscope (Nikon, Tokyo, Japan).

### Transmission electron microscopy

Fresh heart tissues in the margin area of the infarct were cut into less than 1 mm^3^ cubes and rapidly fixed with 2% paraformaldehyde and 2.5% glutaraldehyde at 4 °C for 2 h. The fixed samples were washed three times with PBS. Similarly, H9c2 cells were washed and then fixed by 2% paraformaldehyde and 2.5% glutaraldehyde at 4 °C for 2 h. The cells were collected by gentle scratching gently with a cell scraper. After washed three times, cardiac samples and H9c2 cells were further processed. Copper grids were stained by 2% uranyl acetate, and then embedded with epoxy resin and heated to 60 °C to allow polymerization. Images were recorded by transmission electron microscope (Hitachi-H7650, Tokyo, Japan).

### Mitochondrial membrane potential (ΔΨm)

H9c2 cells were seeded at 1 × 10^5^ cells/mL in a confocal dish. As described above, H9c2 cells were incubated with 5 μM JC-1 probe (Beyotime, Shanghai, China). The depolarization ratio of ΔΨm was determined at an excitation wavelength of 490 nm and 525 nm as JC-1 monomer (green fluorescence) and emission wavelengths of 530 nm and 590 nm as JC-1 aggregate (red fluorescence) in the confocal laser scanning microscopy (Olympus, Tokyo, Japan).

### Real-time quantitative PCR analysis

Total RNA was extracted from H9c2 cells using Trizol reagent (Invitrogen, California, USA) and subjected to reverse transcription. Thereafter, the first cDNA was synthesized using a Revert Aid First Strand cDNA Synthesis kit (Invitrogen, California, USA). Real-time quantitative PCR was carried out using SYBR Premix Ex Taq kit on an ABI PRISM 7500 PCR instrument (Applied Biosystems, New York, USA). The cDNA was denatured by 35 PCR cycles (94 °C, 2 min; 94 °C, 30 s; 61 °C, 30 s; 72 °C, 30 s). GAPDH was the invariant control, and the relative level of mRNA was analyzed by the 2^-ΔΔCt^ method. The sequences for the primers used are listed: PGC-1α: forward, 5’-AGC CAC TAC AGA CAC CGC AC-3′ and reverse, 5’-CCT TTC AGA CTC CCG CTT C-3′ and GAPDH: forward, 5’-GGC AAG TTC AAC GGC ACA G-3′ and reverse, 5’-GCC AGT AGA CTC CAC GAC AT-3′.

### Western blotting

Samples of myocardial tissues from the margin area of the infract were prepared for protein analysis. The heart tissues samples were homogenized in RIPA lysis buffer containing a protease inhibitor cocktail. The same procedures were used for the H9c2 cells. H9c2 cells were collected by scraping with ice-cold RIPA lysis buffer, and the proteins were quantified using BCA protein assay kit. Cell lysates (40 μg of protein) were separated with 12% SDS-PAGE blots and then transferred to the membranes (Millipore, USA). These membranes were blocked with 5% fat-free milk for 2 h and incubated with the following primary antibodies: PGC-1α (Abcam, Cambridge, England), p-AMPK (Cell Signaling Technology, New England, USA), NRF-1 (Abcam, Cambridge, England) and Tfam (Abcam, Cambridge, England) at 4 °C overnight. β-actin was used as a loading control. After washing, the polyvinylidene fluoride membranes were incubated with secondary antibodies (goat anti-rabbit IgG 1:5000 and goat anti-mouse IgG 1:5000) for 2 h. Blots were visualized using with an enhanced chemiluminescence (ECL) detection kit (PIERCE, Massachusetts, USA) and exposure to the X-ray film.

### Statistical analysis

The data were presented as mean ± SD. SPSS 17.0 software (IBM, Armonk, USA) was used for statistical analysis. Student’s t-test or one-way or repeated-measures ANOVA were used to compare values between groups, and *P* less than 0.05 was considered as statistically significant. The analyses were performed by GraphPad Prism software. Xcalibur 2.1 software (Thermo-Fisher Scientific, Bremen, Germany) was used for the qualitative characterization of chemical constituents.

## Results

### Identification of 87 chemical constituents in YQHX

**Fig. 1:**
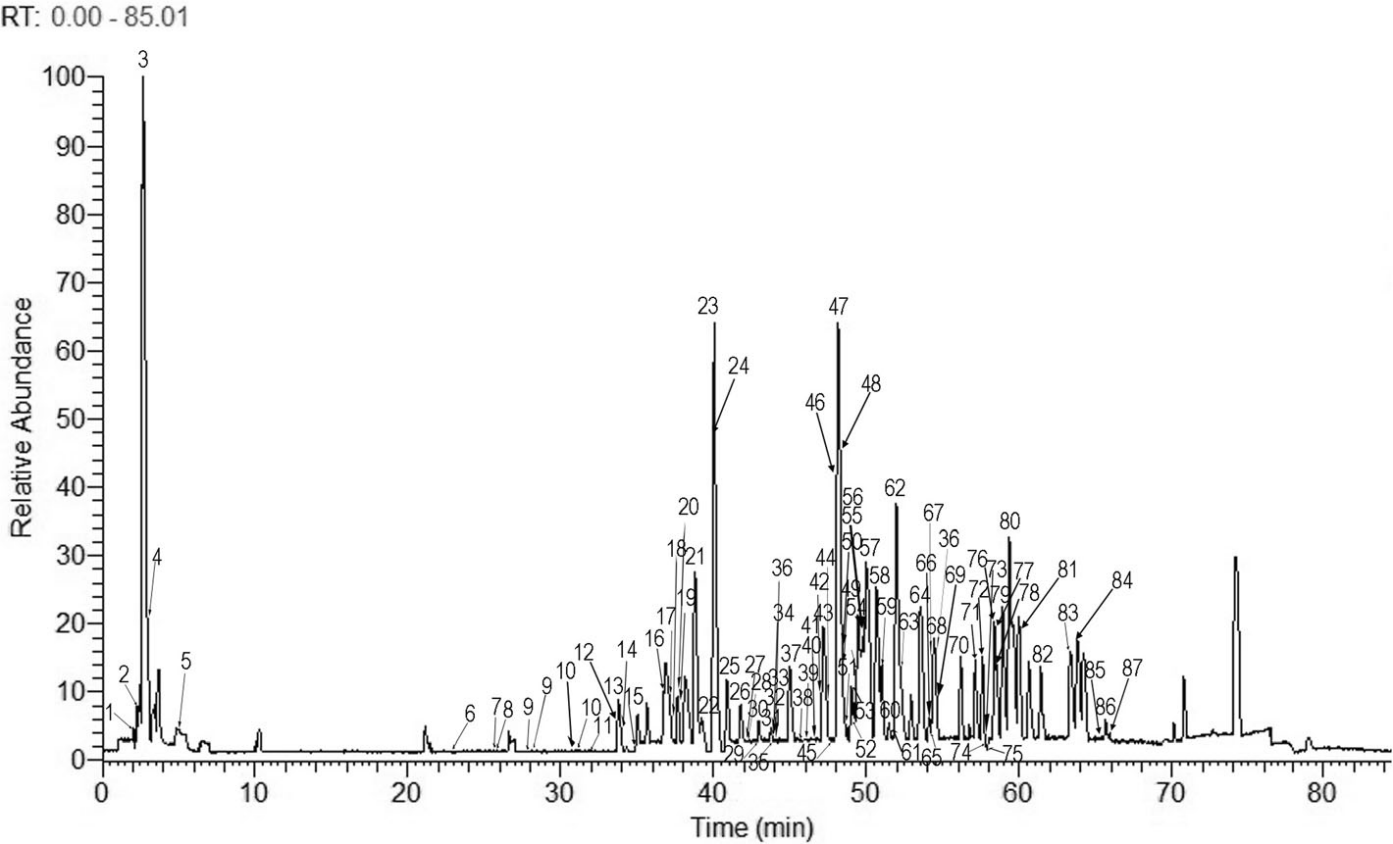
The total ion chromatogram of YQHX obtained in negative ionization mode based on UHPLC-LTQ-Orbitrap-MS

YQHX sample was analyzed with rapid separation using the optimized LC-ESI-MS^n^ method. The total ion chromatogram (TIC) of YQHX sample in negative ionization mode was shown in Fig. 1, and there were 87 peaks in YQHX preparations (Fig. 1 and Additional file 1). For most of the constituents in YQHX, (M-H)^−^ and (M + COOH)^−^ ions were listed in Additional file 1 and consisted of triterpenoid saponins and flavonoids. These data provided valuable information, on the molecular weights and structure of the constituents.

**Fig. 2:**
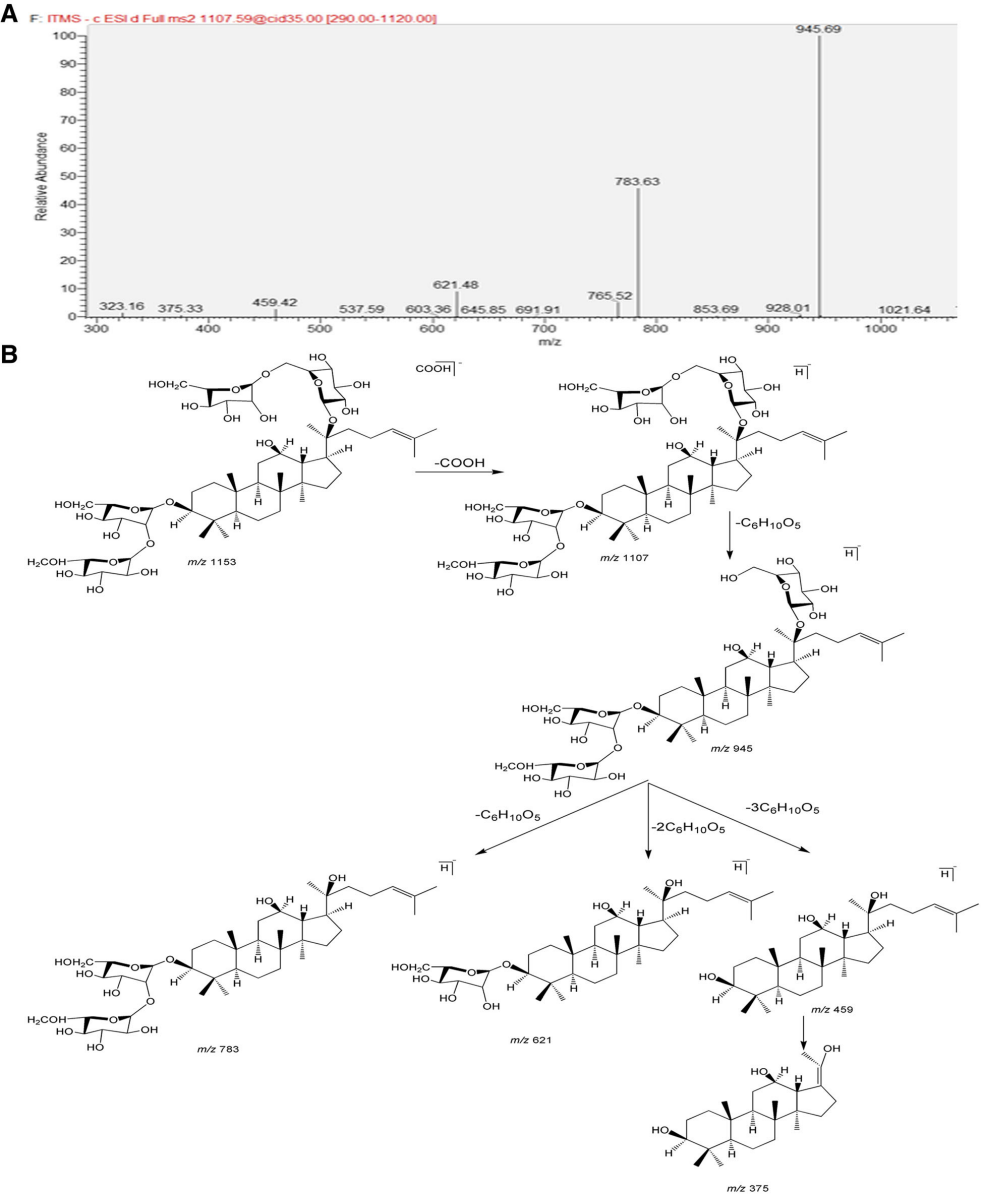
Identification of ginsenoside Rb1 in YQHX preparations. **a** The characteristic fragment ions of ginsenoside Rb1 at *m/z* 945, 783, 621, and 459. **b** The mass spectral fragmentation pathways of ginsenoside Rb1

Taking ginsenoside Rb1 as an example, the structure of chemical components in YQHX was as follows: product 47 (peak 2 in the Fig. 1) displayed quasi-molecular ion peak at *m/z* 1153.59875 (M + COOH)^−^ and 1107.59558 (M-H)^−^ with retention time of 48.13 min and characteristic fragment ions at *m/z* 945, *m/z* 783, *m/z* 621, *m/z* 459 (Fig. 2a), which showed the typical fragmentation characteristics of ginsenoside Rb1. According to the composition analysis, the molecular formula of the compound was C_54_H_92_O_23_ (errors within 1.12 ppm). The fragment ions were produced by the loss of a molecule of glucose, two molecules of glucose, three molecules of glucose, and four molecules of glucose (*m/z* 945 (MH-Glc)^−^, *m/z* 621 (MH-3Glc)^−^and *m/z* 459 (MH-4Glc)^−^), respectively. Therefore, it was identified as ginsenoside Rb1, and the mass spectral fragmentation pathways of ginsenoside Rb1 are shown in Fig. 2b. The structures of the other 86 constituents in YQHX were obtained according to the above method.

### YQHX improved cardiac dysfunction and enhanced PGC-1α expression in MI rat hearts

**Fig. 3:**
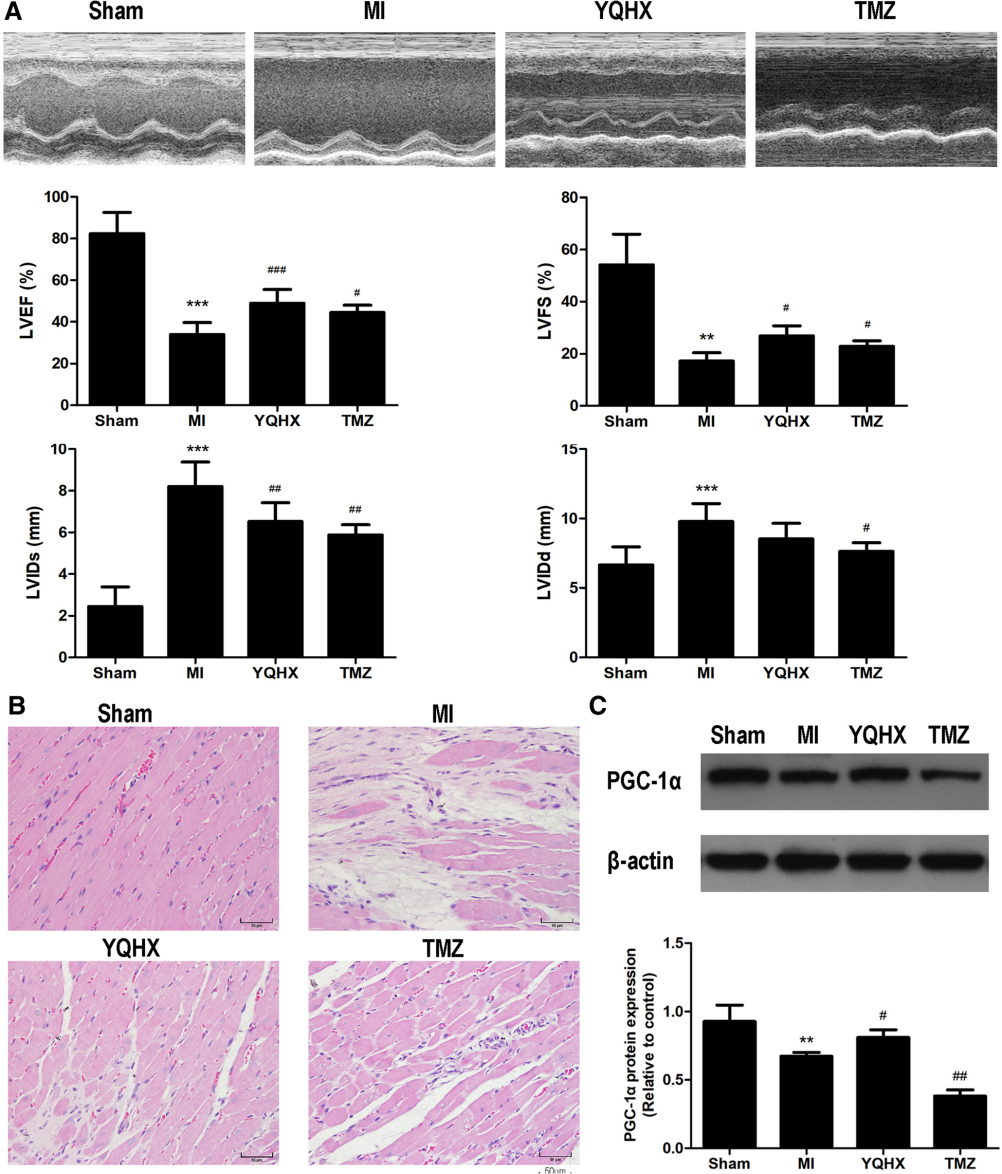
YQHX improved cardiac function and increased PGC-1α protein expression in rats MI model. **a** YQHX and TMZ improved cardiac function and attenuated cardiac remodeling in rats MI model. **b** YQHX and TMZ improved cardiomyocyte architectures in rats MI model. Scale bars = 50 μm. **c** Effects of YQHX and TMZ on PGC-1α protein expression in vivo. Values were represented as mean ± SD (*n* = 8). ^**^P < 0.01, ^***^P < 0.001 versus Sham; ^#^*P* < 0.05, ^##^*P* < 0.01, ^###^*P* < 0.001 versus MI

To determine the effects of YQHX on cardiomyocyte architecture and cardiac function after MI injury, echocardiography analysis showed that YQHX and TMZ significantly improved cardiac dysfunction and reversed cardiac remodeling in MI heart after 4 weeks (Fig. 3a). Specifically, echocardiography showed that YQHX treated groups had a significantly higher LVEF and LVFS, and remarkably lower LVIDs compared with the MI group. However, there was no significant difference in LVIDd between YQHX group and MI group. As shown in Fig. 3b, we observed a high number of inflammatory cell infiltration, enlargement of the intercellular space and extensive edema of cardiomyocytes in rats 4 weeks after MI injury compared to the Sham group. After treatment with YQHX and TMZ, there were numerous changes in the myocardium of rats, including slight inflammatory cell infiltrates and mild edema of cardiomyocytes. Next, we investigated whether YQHX and TMZ affected PGC-1α expression in a rat model of myocardial infarction. TMZ, a pharmacological inhibitor of thiolase and an enzyme involved in a key step of β-oxidation, has been reported to protect against ischemia/reperfusion injury [23]. As shown in Fig. 3c, PGC-1α protein expression in MI group was significantly lower compared with that of Sham group, while YQHX markedly enhanced PGC-1α expression (*P* < 0.05). However, TMZ failed to increase PGC-1α protein expression.

### YQHX protected against MI-induced mitochondrial dysfunction

**Fig. 4:**
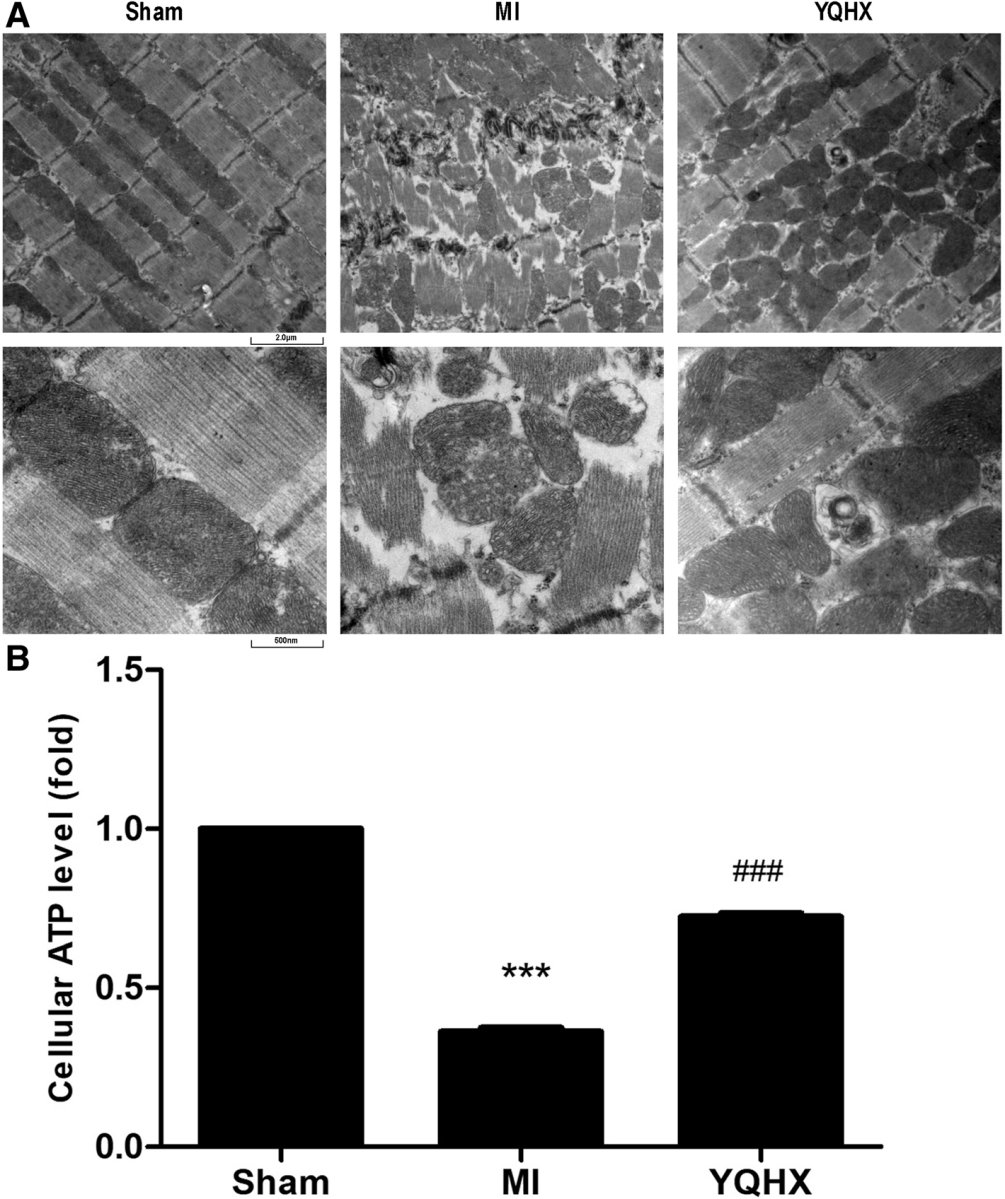
YQHX treatment improved myocardial ultrastructure and mitochondrial function in myocardial infarction rat model. **a** Effect of YQHX treatment on the myocardial ultrastructure in myocardial infarction rat model. Scale bars = 2.0 μm; Scale bars = 500 nm. **b** Effects of YQHX treatment on mitochondrial function in MI rat model. Values were represented as mean ± SD (*n* = 5). ^***^P < 0.001 versus Sham; ^###^*P* < 0.001 versus MI

The sustained energy requirements of the heart require high daily consumption of ATP to sustain the continuous contractile process of the heart [24]. The mitochondria comprise 23% to 32% of the myocellular volume and generate about 90% of the required ATP [24, 25]. We investigated whether the protective effect of YQHX is linked to the mitochondrial functions in rats MI model. As shown in Fig. 4a, the ultrastructure of the myocardium in the Sham group was normal and cardiac muscle fibers were regularly arranged. After MI injury, the myocardial ultrastructure was irregular and that of the mitochondria was disrupted as illustrated by the swelling, rupture, vacuoles and loss of cristae. Interestingly, YQHX treatment improved myocardial ultrastructure during injury and relatively enhanced the proliferation and accumulation of mitochondria. Moreover, chronic myocardial infarction decreased the production of ATP in rats, but YQHX increased ATP production, indicating that YQHX could protect against MI-induced mitochondrial dysfunction (Fig. 4b).

### YQHX enhanced cell viability of H9c2 cells cultivated in normal or ischemic/hypoxic conditions

**Fig. 5:**
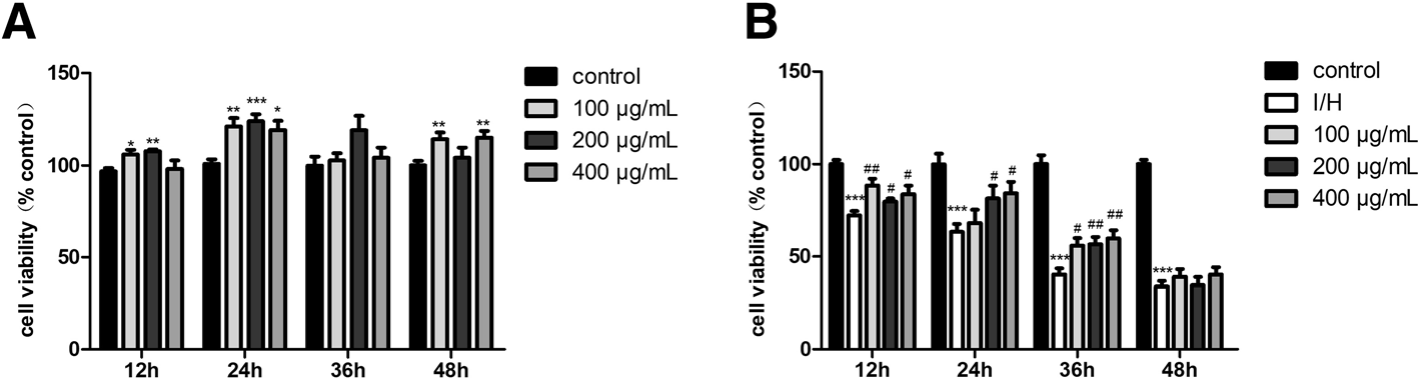
YQHX protected against injury induced by ischemia/hypoxia in H9c2 cardiomyocytes. **a** Effects of YQHX (100–400 μg/mL) on H9c2 cell viability at different time-points in normal conditions. **b** Effects of YQHX (100–400 μg/mL) treatment on H9c2 cell viability subjected to I/H at different time-points. Values were represented as mean ± SD (*n* = 6). ^*^P < 0.05, ^**^P < 0.01, ^***^P < 0.001 versus control; ^#^*P* < 0.05, ^##^*P* < 0.01 versus hypoxia

Based on our previous research [25], YQHX at concentrations of 100–400 μg/mL remarkably increased cell viability induced by hypoxia for 24 h. These concentrations were used in this study for YQHX treatment in normal or hypoxic conditions. Using the CCK8 assay, we found that YQHX significantly enhanced the cell proliferation rate at 12 h, 24 h or 48 h when cells were incubated in normal conditions (Fig. 5a). As shown in Fig. 5b, cell viability of I/H group decreased in a time-dependent manner compared to the control group at 12 h, 24 h, 36 h or 48 h, YQHX at the concentrations of 100–400 μg/mL reversed I/H-induced cell death and significantly increased cell viability at 12 h, 24 h or 36 h. Therefore, the 12 h and 24 h time points were selected for further study.

### YQHX reduced LDH levels induced by hypoxia in H9c2 cells

**Fig. 6:**
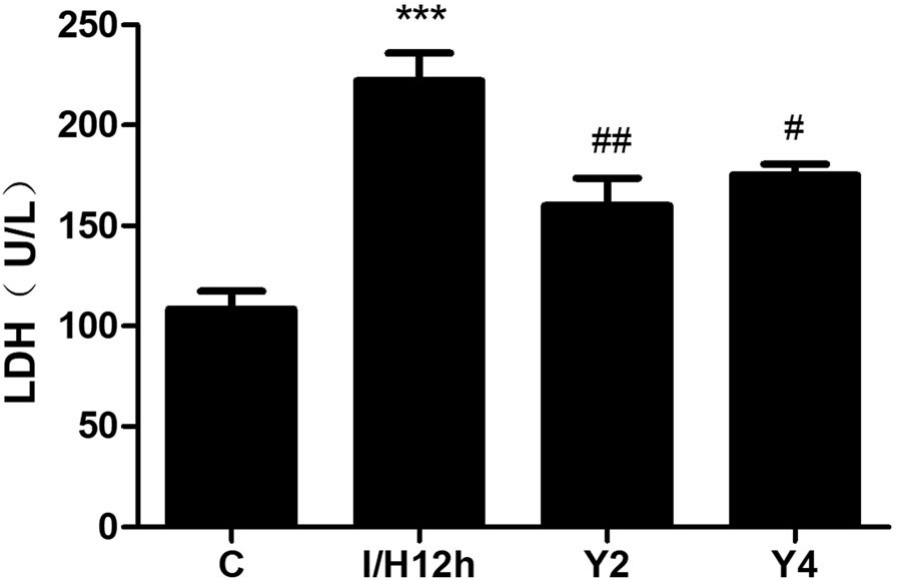
YQHX reversed the level of LDH in H9c2 cells supernatants induced by hypoxia for 12 h. Results were presented as mean ± SD (*n* = 3). ^***^P < 0.001 versus control; ^#^*P* < 0.05, ^##^*P* < 0.01 versus hypoxia

To further assess the cell injury, LDH leakage was tested by LDH assay. The release of LDH in the hypoxia group was significantly elevated compared to the control group at 12 h, YQHX treatment significantly reduced the level of LDH (Fig. 6).

### YQHX improved mitochondrial ultrastructure and reversed the mitochondrial depolarization

**Fig. 7:**
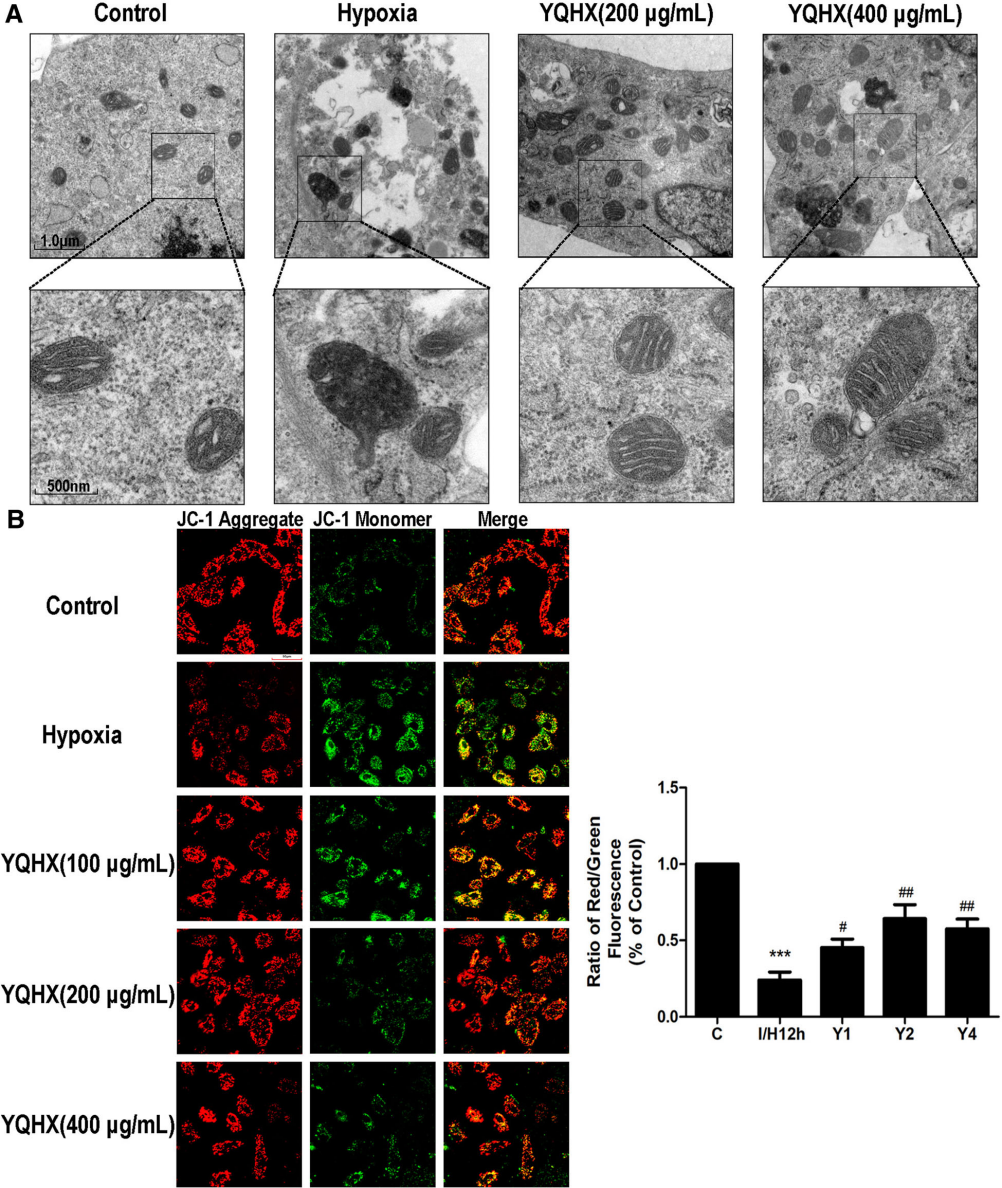
YQHX improved cardiomyocyte mitochondrial ultrastructures and the depolarization of mitochondrial membrane potential. **a** Effect of YQHX on cardiomyocyte mitochondrial ultrastructures in hypoxia-induced H9c2 cells. Scale bars = 1.0 μm; Scale bars = 500 nm. **b** Effect of YQHX treatment on the ΔΨm in H9c2 cells subjected to hypoxia. Scale bars = 50 μm. Data were expressed as mean ± SD (*n* = 4). ^***^P < 0.001 versus control; ^#^*P* < 0.01, ^##^*P* < 0.01 versus hypoxia

Next, we investigated whether YQHX affected mitochondrial ultrastructures in hypoxia-treated H9c2 cells. As shown in Fig. 7a, hypoxia-induced changes in the mitochondrial morphology, including swelling, black matrix and loss of cristae compared to the control. In contrast, YQHX treatment improved the mitochondrial ultrastructures in I/H-induced H9c2 cells.

To further confirm the effect of YQHX on mitochondrial activity, mitochondrial membrane potential (ΔΨm) was observed by confocal laser scanning microscopy in H9c2 cells. ΔΨm was expressed as the fluorescence ratio of red to green. A low ratio represented mitochondrial depolarization. As shown in Fig. 7b, ΔΨm of hypoxia group had a lower ratio than that of the control group, whereas 100–400 μg/mL YQHX treatment reversed the mitochondrial depolarization, especially at the concentration of 200 μg/mL. These data sets indicate that YQHX at the concentration of 200 μg/mL was optimal in protecting the mitochondrial function hypoxia-induced injury in H9c2 cells. Based on these results, the concentration of 200 μg/mL was chosen for the application of YQHX in the present study.

### YQHX increased hypoxia-induced PGC-1α expression in H9c2 cells

**Fig. 8:**
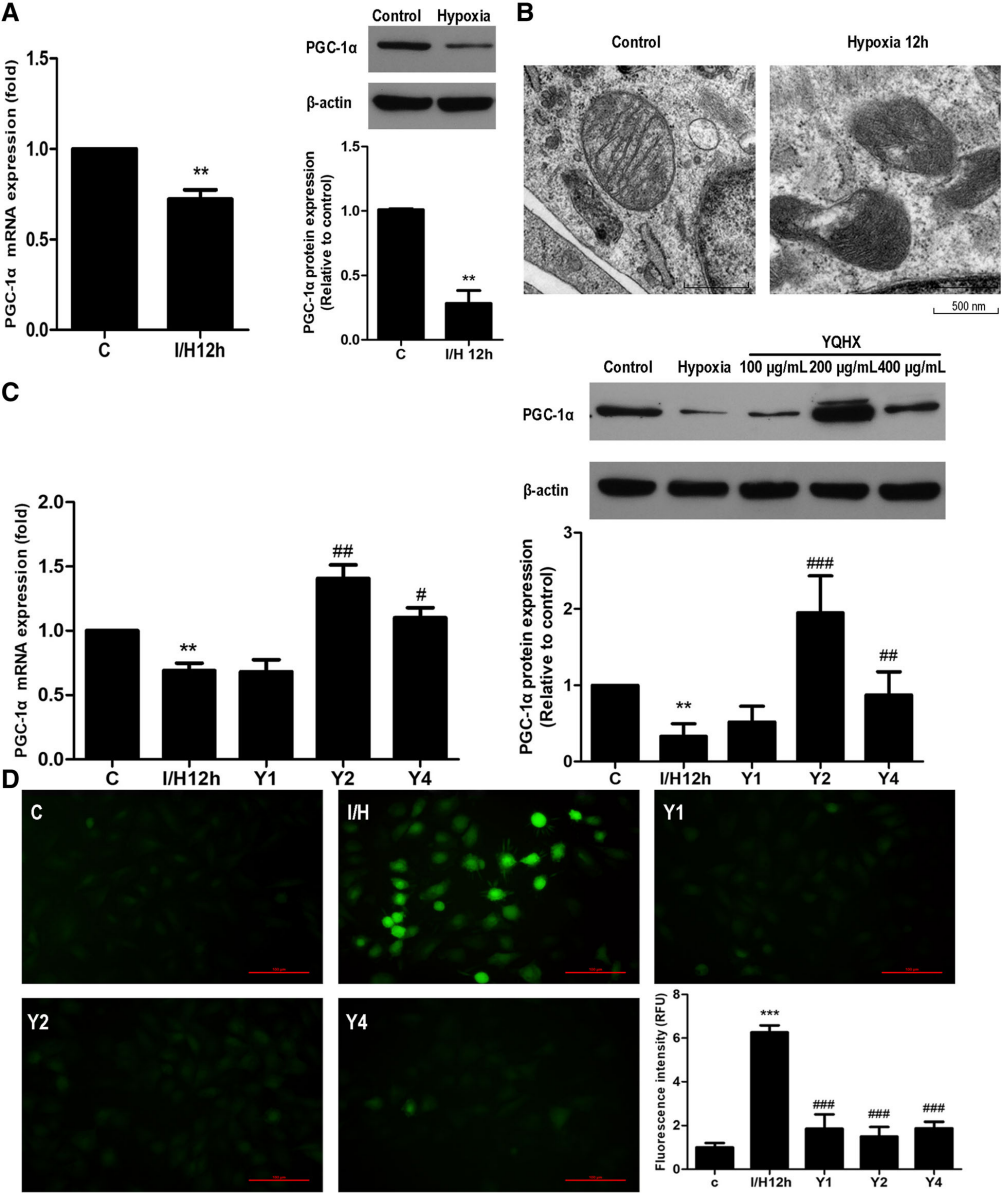
YQHX enhanced PGC-1α mRNA and protein expression and inhibited ROS production in hypoxia-induced H9c2 cells for 12 h. **a** Effects of ischemia/hypoxia on PGC-1α mRNA (left) and protein (right) expression in H9c2 cells. **b** Effect of hypoxia on the mitochondrial structure in hypoxia-treated H9c2 cells. Scale bar = 500 nm. **c** Effects of YQHX treatment on PGC-1α mRNA (left) and protein (right) expression in hypoxia-induced H9c2 cells. **d** Effect of YQHX treatment on the production of ROS in I/H-induced H9c2 cells. Scale bar = 100 μm. The above results were obtained from four independent experiments. Results were presented as mean ± SD (*n* = 4). ^**^P < 0.01, ^***^P < 0.001 versus control; ^#^*P* < 0.05, ^##^*P* < 0.01, ^###^*P* < 0.001 versus hypoxia

We further sought to determine whether hypoxia affected PGC-1α mRNA and protein expression in H9c2 cells. We found that the mRNA and protein levels of PGC-1α were significantly decreased following hypoxia for 12 h (Fig. 8a). Since PGC-1α has been recognized to regulate mitochondrial dysfunction, these results were consistent with the observations that hypoxia was accompanied by mitochondria structural changes, such as black matrix and loss of cristae observed by electron transmission microscopy (Fig. 8b). We further evaluated the effects of YQHX (100–400 μg/mL) on PGC-1α mRNA and protein expression in hypoxia treated H9c2 cells. As shown in Fig. 8c, PGC-1α mRNA expression was significantly decreased in I/H-induced H9c2 cells, whereas 200 μg/mL YQHX treatment remarkably increased the PGC-1α mRNA expression (*P* < 0.01). Similar results were observed for the PGC-1α protein expression in H9c2 cells subjected to 12 h ischemic/hypoxic conditions. These in vitro results were consistent with those obtained from in vivo experiments. Mitochondria are recognized as the major cellular source of ROS. ROS overproduction results in mitochondrial abnormality. Therefore, we investigated the effect of YQHX on ROS production. As shown in Fig. 8d, the production of ROS was increased by hypoxia in H9c2 cells, and 100, 200 and 400 μg/mL YQHX significantly attenuated ROS overproduction.

### YQHX upregulated PGC-1α expression induced by hypoxia via the activation of AMPK phosphorylation

**Fig. 9:**
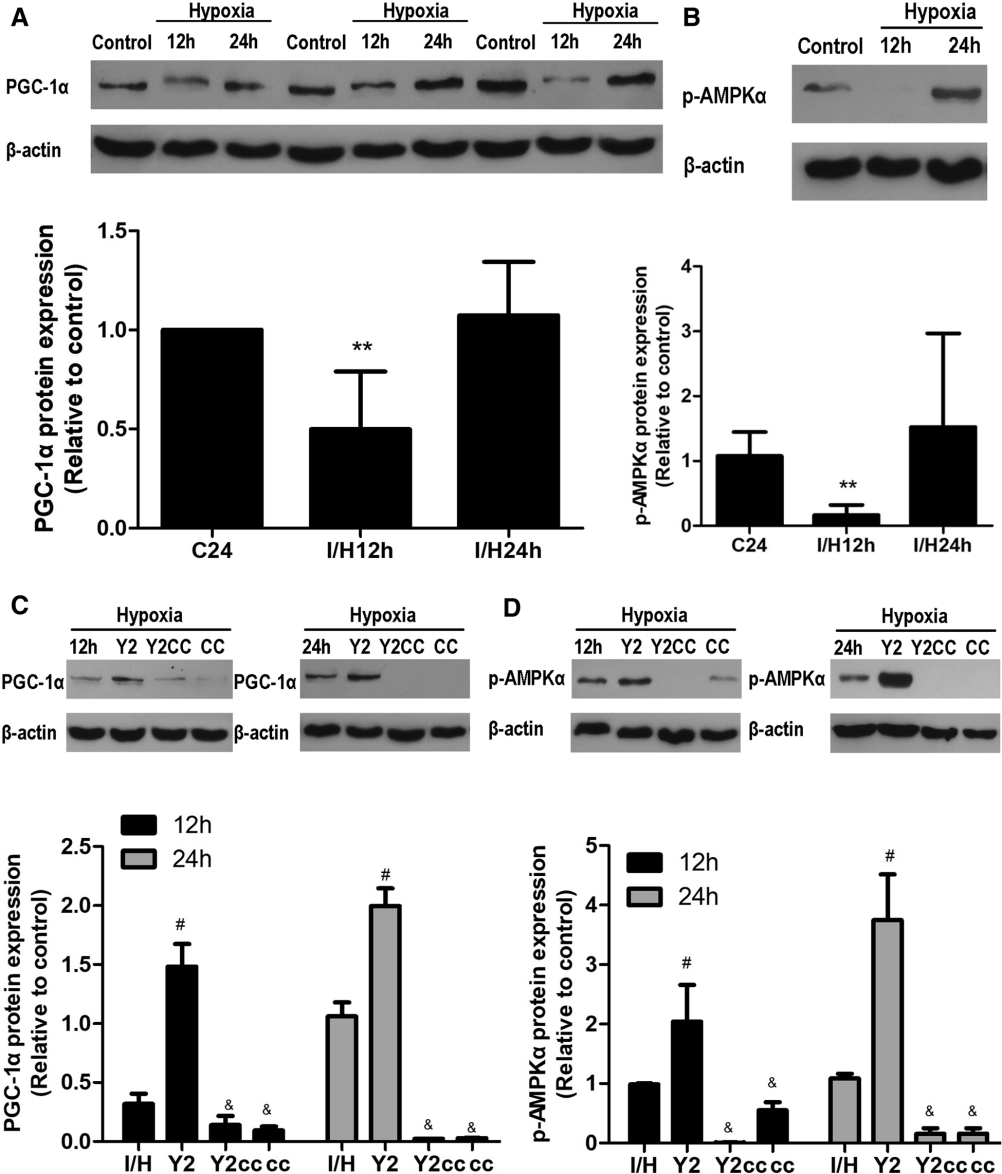
YQHX-induced the expression of PGC-1α through the activation of AMPK phosphorylation in hypoxia-induced H9c2 cells. **a** Effect of YQHX on PGC-1α expression after hypoxia for 12 h and 24 h. **b** Effect of YQHX on AMPK phosphorylation after hypoxia for 12 h and 24 h. **c** YQHX enhanced PGC-1α expression in hypoxia-induced H9c2 cells for 12 h and 24 h, but this effect was completely blocked by 5 μM Compound C. **d** Effect of YQHX and Compound C on AMPK activity after hypoxia for 12 h and 24 h. Values were presented as mean ± SD (*n* = 4). ^**^P < 0.01 versus control; ^#^*P* < 0.05 versus hypoxia; ^&^*P* < 0.05 versus 200 μg/mL YQHX

Studies have demonstrated that the mRNA expression of PGC-1α after exposure to hypoxic conditions for 12 h may differ from 24 h [14, 27]. Hence, we further examined whether exposure of H9c2 cardiomyocytes to 12 h and 24 h hypoxia affected the PGC-1α expression in a similar manner. Based on the above PGC-1α mRNA and protein results in hypoxia-induced H9c2 for 12 h, we further extended hypoxia to 24 h and measured PGC-1α protein expression. Interestingly, compared to the control group, PGC-1α protein expression remarkably decreased at 12 h of hypoxia, but it nearly returned to normal level following 24 h (Fig. 9a). The differences in PGC-1α expression between the control group and hypoxia-induced group at 24 h were not significant. Numerous studies have demonstrated that the upregulation of PGC-1α expression may be directly regulated by AMP-activated protein kinase (AMPK) signaling [9, 14, 27, 28]. To test this possibility, we measured the phosphorylation level of AMPK in hypoxia-induced H9c2 cells at 12 h and 24 h. As shown in Fig. 9b, AMPK phosphorylation at threonine 172 showed an increasing trend in hypoxia-stimulated cells for 24 h, but difference between the control group and hypoxia-induced 24 h group was not significant. This suggests that YQHX-induced activation of PGC-1α expression may be dependent on AMPK activity. We then tested this possibility using Dorsomorphin (Compound C), an inhibitor of AMPK phosphorylation. Treatment with 5 μM Compound C effectively suppressed PGC-1α expression and completely abolished AMPK phosphorylation in hypoxia-stimulated H9c2 cells at 12 h and 24 h (Fig. 9c-d). These data agreed with the results of Ye et al. [14, 26, 27]. Moreover, treatment with YQHX enhanced PGC-1α expression and AMPK phosphorylation in hypoxia-induced H9c2 cells for 12 h and 24 h, but these effects were completely blocked by 5 μM Compound C (Fig. 9c and d). These results further indicate that PGC-1α expression is closely related to AMPK phosphorylation, and that the YQHX-induced activation of PGC-1α expression may be attributed to the activation of AMPK phosphorylation in H9c2 cells subjected to hypoxia.

### YQHX increased the expression of NRF-1 and Tfam in H9c2 cells subjected to hypoxia

**Fig. 10:**
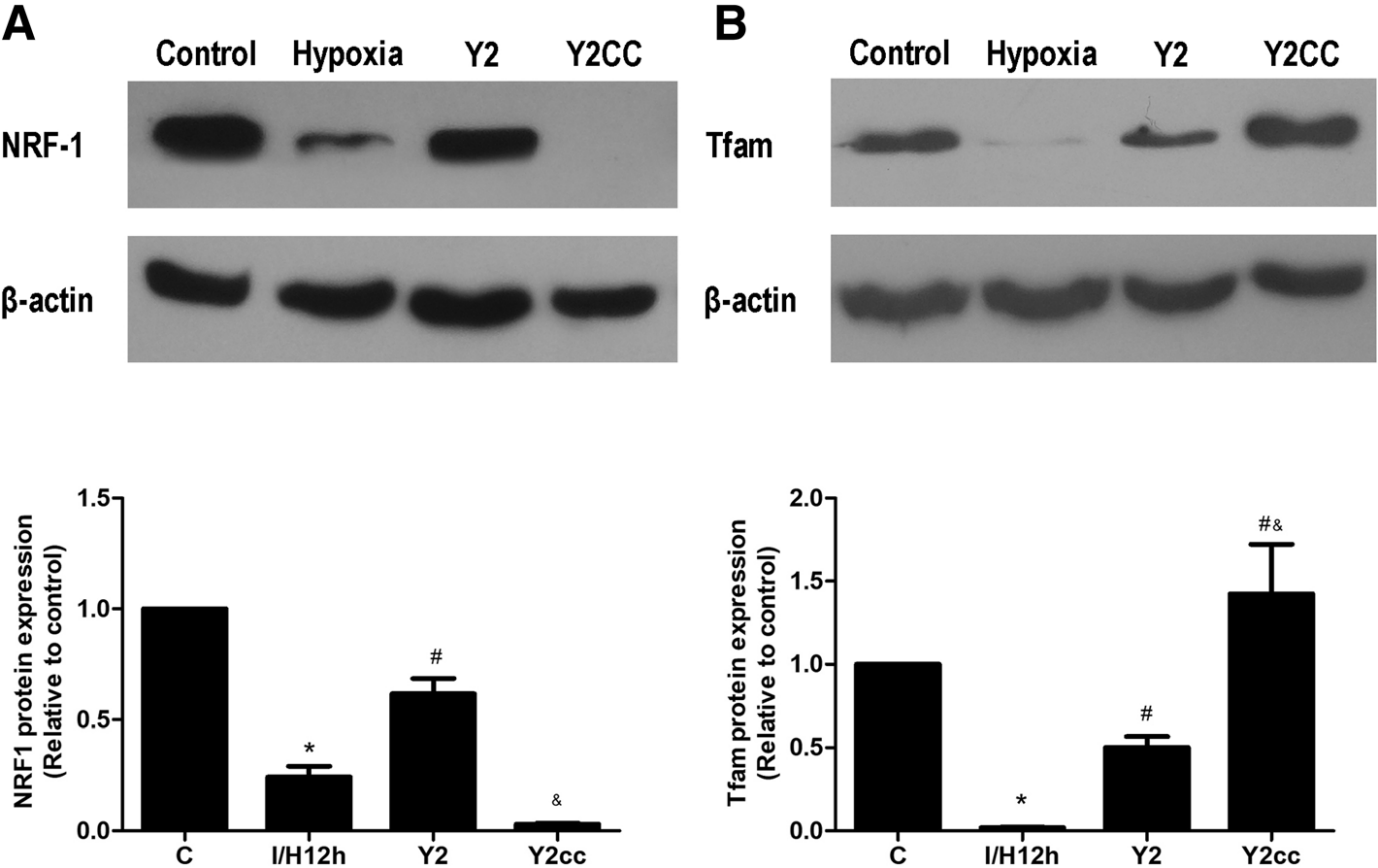
YQHX enhanced the expression of mitochondrial biogenesis molecules induced by hypoxia for 12 h. **a** Effects of YQHX and Compound C on NRF-1 expression in hypoxia-induced H9c2 cells. **b** Tfam expression in hypoxia-induced H9c2 cells. Data were presented as mean ± SD (*n* = 3). ^*^P < 0.05 versus control; ^#^*P* < 0.05 versus hypoxia. ^&^*P* < 0.05 versus 200 μg/mL YQHX. *P* < 0.05 was considered to be statistically significant

Accumulating evidence indicate that PGC-1α is a key controller of mitochondrial function through modulation of the nuclear respiratory factor (NRF-1) and the mitochondrial transcription factor A (Tfam) [21, 29]. A recent study showed that NRF-1 and Tfam expression were inhibited by Compound C in vivo [30]. Hence, we further investigated whether the protective effect of YQHX is related to NRF-1 and Tfam in H9c2 cells subjected to hypoxia. As shown in Fig. 10a, we observed that the expression of NRF-1 was significantly decreased in ischemia/hypoxia group compared with that of the control group (*P* < 0.05), whereas treatment with YQHX markedly increased NRF-1 expression (*P* < 0.05). This effect was completely blocked by Compound C. As shown in Fig. 10b, Tfam expression was lower in ischemia/hypoxia group compared with the control group (*P* < 0.05), while treatment with YQHX significantly increased Tfam expression (*P* < 0.05). Interestingly, this effect of YQHX was not blocked by Compound C. These results illustrate that the protective effects of YQHX on the mitochondrial are partly mediated through AMPK-dependent signaling pathways.

## Discussion

IHD is characterized by the reduction of blood supply to the heart, leading to a series of events such as cardiomyocytes loss and ventricular remodeling all of which lead to heart failure [1]. Therefore, the discovery of effective therapeutic options for IHD is under intense research. Mitochondria are known to be the powerhouses in cardiomyocytes. During ischemia, mitochondria in the cardiomyocytes are the primary subcellular organelles affected by ischemia and are the major source of oxidative stress, leading a series of mitochondrial changes [5]. PGC-1α, a transcriptional coactivator, acts as a master regulator of mitochondrial biogenesis and energy metabolism, and it also decreases oxidative stress [31], and promotes fatty acid oxidation and gluconeogenesis [32]. In this study, we investigated the expression of PGC-1α in the ischemic myocardium. Particularly we investigated the effect of YQHX in cardiomyocytes subjected to hypoxic conditions. We demonstrated that PGC-1α expression was suppressed following ischemic myocardial injury. The major findings of this study are as follows: (1) YQHX treatment might exert cardioprotective effects under ischemic conditions in vitro and vivo. (2) YQHX treatment could upregulate the expression of PGC-1α in MI rats and hypoxia-induced H9c2 cells, which improved the mitochondrial function. (3) YQHX treatment could improve mitochondrial structure and biosynthesis by increasing PGC-1α, NRF-1 and Tfam protein expression in hypoxia-induced H9c2 cells. The up-regulation of PGC-1α, NRF-1, and AMPK phosphorylation expression were completely blocked by AMPK inhibitor Compound C. (4) YQHX contains many active substances such as Astragaloside IV, Calycosin, Ferulic acid, Ginsenoside Rb1, Ginsenoside Rg3, Ginsenoside Rg1 and among others.

Using a myocardial infarction rat model, we firstly measured the LVEF and LVFS levels as representative parameters of cardiac function in rats MI model. These parameters were affected by myocardial infarction, while they were improved by YQHX and TMZ treatment (Fig. 3a). We then observed that YQHX restored the mitochondrial structural integrity and ATP production in the MI myocardium (Fig. 4). Furthermore, we showed that MI decreased the expression of PGC-1α, which was prevented by YQHX treatment. These results were consistent with the observations that PGC-1α was downregulated under chronic hypoxia in pulmonary arterial hypertension [14] and respiratory muscles [33]. Our results supported the hypothesis that YQHX might exert cardioprotection by upregulating PGC-1α expression in vivo. However, the mechanism through which YQHX regulated the PGC-1α expression in the ischemic heart was not previously studied, especially in the cardiomyocytes.

In vitro, we developed the hypoxia injury model in H9c2 cardiomyocytes. Then, we assessed cell viability and LDH release. In the present study, YQHX treatment (100–400 μg/mL) increased cell viability and reduced the LDH release in hypoxia-induced H9c2 cells, suggesting that YQHX has cardio-protective effects on cardiomyocytes during I/H. Mitochondria are recognized as the major cellular source of ROS production and it also act as the powerhouses in cardiomyocytes. Hypoxia induced the downregulation of PGC-1α expression, which may cause ROS overproduction and mitochondrial dysregulation, further exacerbating cardiomyocytes injury. Our data showed that hypoxia up-regulated ROS levels, decreased mitochondrial membrane potential, and disrupted the mitochondrial ultrastructures. Treatment with YQHX decreased ROS levels (Fig. 8d), improved cardiomyocytes mitochondrial ultrastructures and the depolarization of mitochondrial membrane potential, suggesting that YQHX has a protective role on the mitochondrial function in hypoxia-induced cardiomyocytes injury.

PGC-1α is a key precursor of ROS production and extinction [14], which also acts as a powerful regulator of mitochondrial biosynthesis [29]. We also found that hypoxia decreased PGC-1α mRNA and protein expression and damaged mitochondrial ultrastructures in H9c2 cells subjected to hypoxia for 12 h (Fig. 8a-b), while 200 μg/mL YQHX significantly enhanced PGC-1α mRNA and protein expression in hypoxia-induced H9c2 cells. Consistent with our results, hypoxia has been reported to decrease the PGC-1α level in hepatocytes [34]. However, recent studies have demonstrated that PGC-1α expression showed a different response at 12 h and 24 h of hypoxia in pulmonary artery endothelial cells [14]. Therefore, we investigated whether hypoxia for 12 h and 24 h affected the PGC-1α expression in H9c2 cardiomyocytes in a similar manner. Interestingly, PGC-1α protein expression nearly returned to the normal level at 24 h of hypoxia (Fig. 9a), while YQHX markedly upregulated PGC-1α expression compared to 24 h of hypoxia. Furthermore, the effects of hypoxia and YQHX on PGC-1α expression were completely blocked by AMPK inhibitor Compound C (Fig. 9c). AMPK is a well-known serine-threonine kinase, which acts as an intracellular energy sensor. Thus, we subsequently assessed the phosphorylation of AMPK in hypoxia-stimulated H9c2 cells. In agreement of with the changes of PGC-1α expression, phosphorylation of AMPK at Thr172 was significantly decreased at 12 h, but it showed an increasing trend at 24 h of hypoxia compared with the control group (Fig. 9b). Our results indicated that the mechanism of PGC-1α expression occurred via AMPK-mediated phosphorylation, and that YQHX-induced upregulation of PGC-1α expression is closely linked to the activation of AMPK-mediated phosphorylation.

Accumulating evidences indicate that NRF-1 and Tfam are downstream targets of PGC-1α that play a role in mitochondrial biogenesis and respiratory gene expression [34, 35]. Therefore, we hypothesized that YQHX might regulate NRF-1 and Tfam to impact cardio-protection in cardiomyocytes. Indeed, our results showed that YQHX treatment upregulated NRF-1 and Tfam expression in hypoxia-induced H9c2 cells. The upregulation of NRF-1 expression by YQHX was abrogated by Compound C (Fig. 10). However, Compound C in combination with YQHX treatment enhanced Tfam expression in hypoxia-induced H9c2 cells compared with YQHX treatment alone. It is recognized that NRF-1 and Tfam expression can be inhibited by Compound C in vivo [30]. These results indicated that the protective effect of YQHX on H9c2 cells may be orchestrated through multiple targets. They also suggested that the mechanisms of YQHX on PGC-1α expression in hypoxia-induced injury are complex, which point to the possibility that the other unidentified mechanisms contribute to the regulation of PGC-1α expression. For example, silent information regulator of transcription SIRT1, a nutrient sensor through the NAD^+^ dependent histone deacetylation, may deacetylate multiple lysine residues in PGC-1α which promotes mitochondrial fatty acid oxidation [9]. However, the mechanism by which YQHX regulates SIRT1 expression should be further studied.

Protective properties of YQHX have been widely described in the cardiovascular diseases, especially through ECG manifestations and assessment of left ventricular function in patients with myocardial infarction [19]. In rats MI model, our previous studies showed that YQHX regulated the metabolic-related products such as lipids, amino acids and glycolipids [20]. Also, YQHX was reported to ameliorate the cardiac energy metabolism via cross-talk between the LKB1-dependent Notch1 and AMPK after myocardial infarction [21]. However, the chemical constituents in YQHX have not been completely revealed. In this study, we identified 87 constituent compounds in YQHX extracts using the optimized LC-ESI-MS^n^ method. Among the 87 compounds, Astragaloside IV (59), Calycosin (13), Ferulic acid (7), Ginsenoside Rg1 (43), Ginsenoside Rb1 (47), Ginsenoside Rg3 (80), Ginsenoside Rg5 (72), Ginsenoside Rd. (62), and Ginsenoside Re (24) have been reported to have multiple biological activities such as anti-inflammation, anti-apoptosis and anti-oxidative stress, which are related to cardiovascular diseases [36–45]. For example, Astragaloside IV (ASIV, the major component of *Astragalus membranaceus*) was found to alleviate heart failure via regulating glucose and lipid metabolism and to attenuate isoproterenol-induced cardiac hypertrophy by mediating energy biosynthesis [36, 37]. Calycosin (the monomer of *Astragalus membranaceus*) was reported to improve left cardiac function in the myocardial infarction rats model [38]. Ferulic acid (FA, the monomer of *Angelica sinensis*) has been suggested to be a potential treatment for cardiovascular diseases [39]. Ginsenoside Rg1 (G-Rg1, the major constituent of *Panax ginseng* and *Panax notoginseng*) could modulate energy metabolism in rat myocardial ischemia/reperfusion injury [40] and Ginsenoside Rb1 (G-Rb1, the major ingredient of *Panax ginseng*) could decrease the myocardial infarct size and cardiac enzymes [41]. Ginsenoside Rg3 (G-Rg3, the effective components of *Panax ginseng*) could improve cardiac function by attenuating apoptosis and inflammation [42]. Ginsenoside Rg5 (G-Rg5, the monomer of *Panax ginseng*) could decrease cardiomyocytes apoptosis in isoproterenol-induced cardiac ischemia injury [43]. Ginsenoside Rd. (G-Rd, the monomer of *Panax ginseng*) was found to affect ischemia/reperfusion-induced oxidative stress [44]. Ginsenoside Re (G-Re, the monomer of *Panax ginseng*) was also found to have cardioprotective effects against cardiac ischemic and reperfusion injury [45]. The compounds identified in YQHX can be mainly classified into triterpenoid saponins and flavonoids, which mainly belong to *Astragalus membranaceus* and *Panax ginseng*. Our study showed that YQHX treatment significantly enhanced PGC-1α expression in vivo and vitro. Consistent with our results, numerous compounds in YQHX extract were reported to increase PGC-1α levels. For instance, Astragaloside IV could alleviate isoproterenol-induced myocardial hypertrophy by modulating NF-kappaB/PGC-1alpha signaling in energy biosynthesis [37]. Ginsenoside Rb1 was reported to increase basal glucose uptake and to promote browning as evidenced by the increased UCP-1 and PGC-1α mRNA expressions in 3 T3-L1 mature adipocytes [46, 47]. It was also reported that Ginsenoside Rg3 combined with aerobic exercise training enhanced PGC-1α and NRF-2 protein levels in cardiac muscle [48]. These findings indicated that the effects of YQHX on PGC-1α expression in hypoxia-induced H9c2 cells injury might be related to the active components contained in YQHX including Astragaloside IV, Ginsenoside Rb1, Ginsenoside Rg3, and Ginsenoside Rg1 as shown in Fig. 1.

## Conclusions

In summary, the present findings provide evidence that PGC-1α is suppressed in myocardial ischemic injury. Firstly, we confirm that YQHX has 87 chemical constituents using UHPLC-LTQ-Orbitrap-MS. YQHX treatment exerts cardioprotective effects in ischemic conditions both in vivo and vitro. YQHX treatment upregulates the PGC-1α expression in MI rats and improves mitochondrial function. In vitro, YQHX has a pharmacological potential in the treatment of myocardial ischemic injury by upregulating cardiomyocytes PGC-1α, NRF-1, and Tfam expression. The regulation of PGC-1α and NRF-1 expressions were blocked with AMPK inhibitor Compound C. Therefore, the protective mechanism of YQHX on the myocardium through PGC-1α may be due to the regulation of AMPK phosphorylation.

## Additional file

Additional file 1: Structure identification of chemical constituents of YQHX formula by UHPLC-LTQ Orbitrap MS. There were 87 peaks in YQHX preparations. These data provided valuable information, on the molecular weights and structure of the constituents. (DOCX 27 kb)

## Abbreviations

CCK-8: Cell counting kit-8
ECG: Echocardiography
HE: Hematoxylin and eosin
HPLC-DAD-ESI-IT-TOF-MS^n^: High performance liquid chromatography-diode array detection-electrospray ionization-hybrid ion trap-time of flight-mass spectrometry
IHD: Ischemic heart diseases
PGC-1α: Peroxisome proliferator-activated receptor gamma co-activator
qRT-PCR: quantitative real-time polymerase chain reaction
TCM: Traditional Chinese medicine
TEM: Transmission electron microscopy
YQHX: Yiqihuoxue Decoction
