# Supplementary material for: Yiqihuoxue decoction protects against post-myocardial infarction injury via activation of cardiomyocytes PGC-1α expression
Source: BMC Complement Altern Med. 2018 Sep 17;18:253. doi: 10.1186/s12906-018-2319-1 (PMC6142634; doi:10.1186/s12906-018-2319-1)
Supplement: Supplementary file 1 — Structure identification of chemical constituents of YQHX formula by UHPLC-LTQ Orbitrap MS. There were 87 peaks in YQHX preparations. These data provided valuable information, on the molecular weights and structure of the constituents. (DOCX 27 kb) [file 12906_2018_2319_MOESM1_ESM.docx]

**Structure identification of chemical constituents of YQHX formula by UHPLC-LTQ Orbitrap MS**

| No. | _RT_/min | Molecular formula | (+COOH)  Observed mass(*m/z*) | Calculated mass(*m/z*) | Mass accuracy(ppm) | (-)-ESI-MS/MS (*m/z*) | Identification |
| --- | --- | --- | --- | --- | --- | --- | --- |
| 1 | 2.29 | C_10_H_12_O_2_ | 209.08092 | 209.08083 | -0.43 | MS2:190.91;209.02 | Isoeugenol |
| 2 | 2.58 | C_5_H_10_O_5_ | 195.05008 | 195.04992 | -0.82 | MS2:176.96;159.01;140.97 | Xylose |
| 3 | 2.7 | C_12_H_22_O_11_ | 341.10764 | 341.10783 | 0.56 | MS2: 179.02;220.93;281.10;323.04 | Maltose |
| 4 | 2.93 | C_18_H_32_O_16_ | 503.16119 | 503.16066 | -1.05 | MS2: 485.44；459.44；443.33 | Umbelliferose |
| 5 | 5.26 | C_9_H_12_N_2_O_6_ | 243.06223 | 243.06116 | -4.4 | MS2:199.98;225.20;174.88;152.97 | Uridine |
| 6 | 22.96 | C_17_H_14_O_7_ | 375.06961 | 375.07105 | 3.84 | MS2:200.87;178.97;271.05;317.20 | 3,3'-Dimethylquercetin |
| 7 | 25.74 | C_10_H_10_O_4_ | 193.05007 | 193.04953 | -2.8 | MS2: 148.89;178.01;192.37;133.99 | Ferulic acid |
| 8 | 25.86 | C_20_H_22_O_7_ | 419.1344 | 419.13365 | -1.79 | MS2: 241.15;152.04;349.22;399.24;285.13 | Hyuganin D |
| 9 | 27.73 | C_42_H_74_O_16_ | 879.49683 | 879.49479 | -2.32 | MS2: 833.47;861.05;760.12;671.76 | Notoginsenoside J |
| 9 | 28.3 | C_42_H_74_O_16_ | 879.49622 | 879.49479 | -1.63 | MS2: 833.45;773.27;653.43 | Notoginsenoside J |
| 10 | 30.42 | C_16_H_18_O_9_ | 353.08786 | 353.0867 | -3.29 | MS2: 191.06.;179.05;135.01;234.05;284.68;309.14 | 3-O-Caffeoylquinic acid |
| 10 | 31.31 | C_17_H_20_O_9_ | 367.10336 | 367.10235 | -2.75 | MS2: 172.97;203.76;299.02;338.12;154.85; | 3-O-Feruloylquinic acid |
| 11 | 31.91 | C_42_H_74_O_15_ | 863.50128 | 863.49987 | -1.63 | MS2: 863.50;695.18;10007.54;1277.66;1516.90;1960.03;215.00 | Quinquenoside L9 |
| 12 | 33.75 | C_22_H_22_O_10_ | 491.11954 | 491.1184 | -2.32 | MS2: 283.04;327.97;444.75;268.03;207.12;166.97 | 7,5'-Hydroxy-3'-methoxyisoflavone 7-O-glucoside |
| 13 | 33.79 | C_16_H_12_O_5_ | 283.0611 | 283.06009 | -3.57 | MS2:267.99;283.29;255.10;238.64 | Calycosin |
| 14 | 33.91 | C_27_H_30_O_16_ | 609.14551 | 609.14501 | -0.82 | MS2: 413.30;431.09;539.12;584.97;258.97;215.01; | Kaempferol 3,4'-di-O-beta-D-glucopyranoside |
| 15 | 34.81 | C_42_H_70_O_15_ | 859.46967 | 859.46857 | -1.28 | MS2: 815.37;724.66;653.59;567.02;491.60 | Floralginsenoside F |
| 16 | 36.75 | C_53_H_90_O_23_ | 1139.58459 | 1139.58439 | -0.18 | MS2: 1148.27;1117.02;1088.76 | Floralginsenoside P |
| 17 | 37.28 | C_16_H_18_O_9_ | 353.08783 | 353.0867 | -3.2 | MS2: 172.85;217.07;284.97;335.18;134.97 | Scopolin |
| 18 | 37.34 | C_17_H_14_O_5_ | 297.07666 | 297.07575 | -3.06 | MS2:284.08;255.18;230.86;172.91 | Afrormosin |
| 19 | 37.87 | C_15_H_10_O_4_ | 299.05606 | 299.05501 | -3.51 | MS2:283.97;231.00;178.59;144.99 | Chrysophanol |
| 20 | 37.87 | C_16_H_12_O_6_ | 299.05609 | 299.05501 | -3.61 | MS2:284.01;254.70;230.75;207.14;157.80 | Kaempferide |
| 21 | 38.75 | C_47_H_80_O_18_ | 977.53186 | 977.53157 | -0.3 | MS2: 931.70;830.90;769.63;599.55 | Ginsenoside R1 |
| 22 | 39.31 | C_41_H_70_O_15_ | 847.46863 | 847.46857 | -0.07 | MS2; 799.55;739.64;825.81;637.60;475.49 | Floralginsenoside C |
| 23 | 40.05 | C_42_H_72_O_14_ | 845.48944 | 845.48931 | -0.15 | MS2:799.47;637.56;709.28;842.36 | Ginsenoside Rf |
| 24 | 40.05 | C_48_H_82_O_18_ | 991.5484 | 991.54722 | -1.19 | MS2: 945.66;855.41;793.65;591.48;341.11 | Ginsenoside Re |
| 25 | 40.88 | C_23_H_24_O_11_ | 475.12488 | 475.12348 | -2.95 | MS2: 267.08;252.20;293.72;351.16;473.45 | 7,2'-Dihydroxy-3',4'-dimethoxyisoflavone 7-O-glucoside |
| 26 | 41.75 | C_11_H_12_O_3_ | 237.07666 | 237.07575 | -3.84 | MS2:192.97;102.91;218.68;153.06 | Myristicin |
| 27 | 42.41 | C_48_H_80_O_19_ | 1005.52722 | 1005.52648 | -0.74 | MS2: 959.59;869.10;797.03;562.41 | Notoginsenoside G |
| 28 | 42.44 | C_56_H_92_O_25_ | 1163.58435 | 1163.58439 | 0.03 | MS2: 1151.89;1013.73;855.79 | Malonylginsenoside Rc |
| 29 | 42.73 | C_54_H_92_O_24_ | 1169.59668 | 1169.59495 | -1.48 | MS2: 1154.18;1035.47;785.46 | Notoginsenoside A |
| 30 | 42.96 | C_23_H_26_O_10_ | 507.15115 | 507.1497 | -2.86 | MS2: 460.99;488.44;438.96;421.24 | Methylnissolin 3-O-glucoside |
| 31 | 43.68 | C_41_H_70_O_13_ | 815.47974 | 815.47874 | -1.23 | MS2: 637.43;678.83;607.54;769.45;475.51 | Ginsenoside F3 |
| 32 | 43.74 | C_22_H_26_O_8_ | 463.16135 | 463.15987 | -3.2 | MS2: 301.15;344.27;395.08;443.12;227.05 | (+)-Lirioresinol B |
| 33 | 43.74 | C_23_H_28_O_10_ | 463.16116 | 463.15987 | -2.79 | MS2: 443.12;419.34;448.61;427.48 | Isomucronulator 7-O-glucoside |
| 34 | 43.85 | C_44_H_74_O_15_ | 887.50031 | 887.49987 | -0.5 | MS2: 841.50;781.51;637.52;465.37;325.25 | Vina-ginsenoside R1 |
| 35 | 43.85 | C_45_H_76_O_17_ | 887.50055 | 887.49987 | -0.77 | MS2: 841.50;781.51;637.52;465.37;325.25 | Oleifolioside A |
| 36 | 44 | C_50_H_84_O_19_ | 1033.55847 | 1033.55778 | -0.67 | MS2: 1015.16;896.79;809.41 | Quinquenoside III |
| 37 | 44.97 | C_16_H_12_O_5_ | 283.06107 | 283.06009 | -3.46 | MS2:267.98;283.21;255.20;221.40 | (-)-Maackiain |
| 38 | 45.76 | C_47_H_78_O_19_ | 991.51251 | 991.51083 | -1.69 | MS2: 923.09;745.61;619.66 | Astragaloside VII |
| 39 | 46.18 | C_59_H_100_O_27_ | 1285.64331 | 1285.6423 | -0.79 | MS2: 1264.88;1089.85;942.57;704.70;397.18 | Notoginsenoside R4 |
| 40 | 46.76 | C_41_H_70_O_14_ | 831.47449 | 831.47366 | -1 | MS2: 785.68;750.21;657.35 | Ginsenoside Rg8 |
| 41 | 46.76 | C_42_H_72_O_16_ | 831.47467 | 831.47366 | -1.21 | MS2: 785.68;750.21;657.35 | Floralginsenoside A |
| 42 | 46.97 | C_59_H_100_O_27_ | 1285.64264 | 1285.6423 | -0.26 | MS2: 1191.65;961.20;806.56 | Ginsenoside Ra3 |
| 43 | 47.18 | C_42_H_72_O_14_ | 845.48987 | 845.48931 | -0.66 | MS2:799.47;637.56;709.28 | Ginsenoside Rg1 |
| 44 | 47.75 | C_36_H_62_O_10_ | 699.43188 | 699.4314 | -0.69 | MS2: 653.52;631.08;575.17;529.20;432.27 | Notoginsenoside R8 |
| 45 | 47.83 | C_12_H_14_O_4_ | 221.08182 | 221.08083 | -4.48 | MS2:203.05;177.02;149.08;88.93 | Dillapiole |
| 46 | 48.04 | C_10_H_12_O | 177.09183 | 177.091 | -4.69 | MS2:177.16;148.95;132.92 | Estragol |
| 47 | 48.13 | C_54_H_92_O_23_ | 1153.59875 | 1153.60004 | 1.12 | MS2: 945; 621; 459 | Ginsenoside Rb1 |
| 48 | 48.33 | C_41_H_70_O_13_ | 815.47943 | 815.47874 | -0.85 | MS2: 769.61;69682;638.13 | Chikusetsusaponin L8 |
| 49 | 48.72 | C_56_H_94_O_24_ | 1195.60742 | 1195.6106 | 2.66 | MS2: 1150.80;1109.21;944.71 | Quinquenoside R1 |
| 50 | 48.72 | C_57_H_94_O_26_ | 1193.59497 | 1193.59495 | -0.02 | MS2: 1147.25;766.74 | Malonyl-ginsenoside Rb1 |
| 51 | 48.93 | C_58_H_98_O_26_ | 1209.62439 | 1209.62625 | 1.54 | MS2: 1150.72;1058.70 | Ginsenoside Ra1 |
| 52 | 49.02 | C_53_H_90_O_22_ | 1123.59021 | 1123.58947 | -0.66 | MS2: 1055.23;960.77;829.91;722.73 | Ginsenoside Rb2 |
| 53 | 49.21 | C_41_H_70_O_13_ | 815.47992 | 815.47874 | -1.45 | MS2: 769.57;796.57;747.18;653.34;559.45 | Ginsenoside R2 |
| 54 | 49.44 | C_42_H_72_O_13_ | 829.49463 | 829.49439 | -0.29 | MS2: 783.54;738.88;792.60 | Ginsenoside F2 |
| 55 | 49.83 | C_47_H_74_O_17_ | 955.48999 | 955.4897 | -0.3 | MS2: 793.61;835.58;937.27;731.62;613.52 | Trojanoside I |
| 56 | 49.83 | C_48_H_76_O_19_ | 955.48999 | 955.4897 | -0.3 | MS2: 793.52;523.51;835.69 | Chikusetsusaponin V |
| 57 | 50 | C_36_H_62_O_9_ | 683.43652 | 683.43648 | -0.06 | MS2: 637.49;647.53;559.13;475.46;437.37 | Ginsenoside Rh1 |
| 58 | 50.64 | C_36_H_62_O_9_ | 683.43652 | 683.43648 | -0.06 | MS2: 637.50;665.75;615.85;583.62;559.17 | (20R)-Ginsenoside RH1 |
| 59 | 50.99 | C_41_H_68_O_14_ | 829.45898 | 829.45801 | -1.17 | MS2:783.46;621.41;489.34 | Astragaloside IV |
| 60 | 51.46 | C_47_H_74_O_18_ | 925.48022 | 925.47914 | -1.17 | MS2: 569.48;655.43;793.52;901.49;497.65 | Pseudo-ginsenoside RT1 |
| 61 | 51.52 | C_41_H_68_O_14_ | 829.45929 | 829.45801 | -1.54 | MS2:783.49;713.14;806.30 | Astragaloside III |
| 62 | 51.93 | C_48_H_82_O_18_ | 991.54675 | 991.54722 | 0.47 | MS2: 945.74;907.25;775.54;378.88 | Ginsenoside Rd |
| 63 | 52.14 | C_17_H_12_O_6_ | 357.06097 | 357.06049 | -1.34 | MS2:313.10;339.25;269.02;225.03 | Acicerone |
| 64 | 53.54 | C_48_H_82_O_18_ | 991.54791 | 991.54722 | -0.7 | MS2: 945.50;855.78;971.71 | Notoginsenoside K |
| 65 | 54.24 | C_43_H_72_O_15_ | 873.48511 | 873.48422 | -1.02 | MS2: 825.47;765.49;603.63;473.71 | Vina-ginsenoside R2 |
| 66 | 54.37 | C_16_H_12_O_4_ | 267.06616 | 267.06518 | -3.67 | MS2:252.01;207.16;165.29;132.28 | Formononetin |
| 67 | 54.41 | C_43_H70O_15_ | 871.4693 | 871.46857 | -0.84 | MS2: 825.48;765.52;603.48;489.28 | Trojanoside A |
| 68 | 55.14 | C_47_H_80_O_17_ | 961.53699 | 961.53665 | -0.35 | MS2: 893.04;824.60;527.19 | Notoginsenoside Fe |
| 36 | 55.17 | C_50_H_84_O_19_ | 1033.55811 | 1033.55778 | -0.32 | MS2: 964.60; 988.01; 917.28 | Quinquenoside III |
| 69 | 55.29 | C_48_H_78_O_18_ | 941.51141 | 941.51044 | -1.03 | MS2: 923.63;795.55;615.53;525.55;457.40 | Soyasaponin I |
| 70 | 56.15 | C_43_H_70_O_15_ | 871.46973 | 871.46857 | -1.33 | MS2: 825.68;765.52;603.22 | Isoastragaloside II |
| 71 | 57.11 | C_42_H_70_O_12_ | 811.48444 | 811.48383 | -0.75 | MS2: 765.61;742.98;616.62;513.35;457.36 | (20E)-Ginsenoside F4 |
| 72 | 57.56 | C_42_H_70_O_12_ | 811.48505 | 811.48383 | -1.5 | MS2: 765.67;802.06;710.85;584.62;465.15 | Ginsenoside Rg5 |
| 73 | 57.7 | C_42_H_72_O_13_ | 829.49329 | 829.49439 | 1.33 | MS2: 783.42;719.93;621.48;459.53 | Ginsenoside Rg2 |
| 74 | 57.79 | C_43_H_70_O_15_ | 871.46887 | 871.46857 | -0.34 | MS2: 825.55;853.49;765.53;603.48;547.70 | Astragaloside II |
| 75 | 57.91 | C_47_H_80_O_17_ | 961.53766 | 961.53665 | -1.05 | MS2:915.77;800.79;716.60;405.22 | Gypenoside IX |
| 76 | 58.2 | C_47_H_74_O_18_ | 925.48016 | 925.47914 | -1.1 | MS2: 549.46;613.45;745.50;881.29;455.50 | Araloside A |
| 77 | 58.26 | C_12_H_12_O_3_ | 203.07101 | 203.07027 | -3.64 | MS2: 159.95;174.03;203.06;146.89 | 3-Butylidene-7-hydroxyphthalide |
| 78 | 58.37 | C_36_H_60_O_8_ | 665.42706 | 665.42592 | -1.71 | MS2: 619.50;646.19;573.57;501.20;432.65 | Ginsenoside Rh4 |
| 79 | 58.87 | C_36_H_60_O_8_ | 665.42645 | 665.42592 | -0.8 | MS2: 619.49;597.33;527.76;484.98;380.23 | Ginsenoside Rk3 |
| 80 | 59.34 | C_42_H_72_O_13_ | 829.49426 | 829.49439 | 0.16 | MS2: 783.54;810.94 | Ginsenoside Rg3 |
| 81 | 59.95 | C_45_H_72_O_16_ | 913.47992 | 913.47914 | -0.85 | MS2: 867.66;807.72;717.88;489.53 | Astragaloside I |
| 82 | 61.37 | C_45_H_72_O_16_ | 913.47998 | 913.47914 | -0.92 | MS2: 867.55;807.61;686.84;353.42 | Isoastragaloside I |
| 83 | 63.35 | C_42_H_70_O_12_ | 811.48431 | 811.48383 | -0.59 | MS2: 765.53;792.87;674.95;535.14;465.10 | Ginsenoside Rg6 |
| 84 | 63.8 | C_42_H_70_O_12_ | 811.4837 | 811.48383 | 0.16 | MS2: 765.56;742.72;470.46 | Ginsenoside Rk1 |
| 85 | 65.25 | C_26_H_30_O_5_ | 467.20538 | 467.20642 | 2.23 | MS2: 397.22;353.32;419.34;275.13;207.19 | Xanthoangelol G |
| 86 | 65.62 | C_36_H_62_O_8_ | 667.44238 | 667.44157 | -1.21 | MS2:621.68;649.27;599.47;511.15;459.44 | 20(R)-Ginsenoside Rh2 |
| 87 | 65.91 | C_36_H_62_O_8_ | 667.44263 | 667.44157 | -1.59 | MS2: 621.58;648.49;598.83;511.65;459.48 | Notoginsenoside R7 |
